# Moving as We Age: Effects of Physical Activity Programmes on Older Adults—An Umbrella Review

**DOI:** 10.3390/geriatrics10040098

**Published:** 2025-07-23

**Authors:** Ruth D. Neill, Louise Bradley, Roger O’Sullivan

**Affiliations:** 1 Institute of Public Health, D08 NH90 Dublin, Ireland; louise.bradley@publichealth.ie; 2 The Bamford Centre, Ulster University, Coleraine BT52 1SA, UK; roger.osullivan@publichealth.ie

**Keywords:** physical activity, older adults, programme, rapid umbrella review, ageing

## Abstract

**Background**: This paper aims to conduct an umbrella review of the effects of physical activity programmes for older adults (aged 70 and above). **Methods**: Comprehensive literature searches were conducted in MEDLINE, PubMed, EMBASE, PsychINFO, and Cochrane Library databases for English SRs. Inclusion criteria were systematic reviews that included randomised controlled trials examining physical activity interventions in older adults. The data extracted were participant characteristics, physical activity interventions, and outcomes examined. A synthesis of results was conducted using the PRISMA guidelines, and the quality of the studies was assessed using the Assessment of Multiple Systematic Reviews-2 (AMSTAR-2). **Results**: Ten systematic reviews on 186 research articles were included. The AMSTAR-2 revealed that 4 out of 10 reviews were of high quality and 1 out of 10 were of moderate quality. The study samples in each systematic review ranged from 6 to 1254 participants. The total overall sample size for the 10 included studies was 22,652 participants. Across the included reviews, there was mixed evidence on whether physical activity interventions could improve outcomes in older adults across various settings. **Conclusions**: Sample sizes and findings in each included systematic review varied. The findings of this review emphasise the importance of physical activity as a vital component in maintaining and enhancing health, as well as combating poor health as we age. It also highlights the need for a deeper understanding of the specific physical activity requirements for those aged 70 and above. Future systematic reviews may focus on streamlined reporting of dosing of physical activity and specific intervention types, such as group versus single.

## 1. Introduction

Today, people live longer than they did in previous decades, thanks to advances in healthcare and improved survival rates. By 2050, one in four people will be over 65 [1], and by 2074, the number of people over 80 is projected to triple [2]. As the population ages, quality of life, mental and physical health, and cognitive function are important factors. The growing ageing population has critical societal and economic considerations, particularly regarding public health. The shift in population ageing began in higher-income countries, such as Japan, and is now being experienced in low- and middle-income countries as well [1,3]. The World Health Organisation and the UN Decade of Ageing have emphasised the importance of active ageing by promoting healthy ageing, lifelong learning, financial security, and active participation in society. However, maintaining physical and mental health as we age can bring challenges [3].

Physical activity is a vital component of healthy ageing. Evidence has established that regular physical activity is essential for maintaining the physical and mental health of older adults [4,5,6,7]. Physical activity can be a protective factor for non-communicable diseases such as diabetes, cardiovascular diseases, and certain cancers [8,9]. The health benefits can also reduce the risk of falls, improving cognitive function and immune response [10,11]. Older adults must adhere to the recommended physical activity guidelines for optimal effects. The World Health Organisation [12] recommends at least 150–300 min of moderate-intensity or 75–150 min of vigorous-intensity activity per week for older adults. As the population ages, physical inactivity levels generally increase in men and women [12]. It is estimated that globally, 42% of older adults are not meeting the recommended levels of physical activity, and inactivity levels are twice as high in high-income countries compared to low-income countries [12]. Previous research has indicated that as we age, several issues can develop that may impact one’s level of physical activity and mental health, fragility, cognitive abilities, socioeconomic status, and location/home setting [3,8,13,14,15,16].

Several systematic reviews and meta-analyses have investigated physical activity interventions across several populations [17,18,19,20]. To our knowledge, no rapid umbrella review on the effects of physical activity programmes for older adults (aged 70+) has been published. Therefore, to synthesise and evaluate the available evidence, we conducted an umbrella review of systematic reviews that assessed physical activity interventions in older adults.

## 2. Materials and Methods

### 2.1. Structure of Umbrella Review

An umbrella review is a systematic collection and evaluation of several systematic reviews and meta-analyses [21]. To support our evidence on associations, we sought to collect information from systematic reviews that involved randomised controlled trials. We compared results from each systematic review and meta-analysis, which provided quantitative data. This umbrella review adhered to the JBI guidelines where applicable [22], the Preferred Reporting Items for Systematic Reviews (PRISMA) guidelines [23] and the protocol was registered with the International Prospective Register of Systematic Reviews (Prospero registration number: CRD42024521063).

### 2.2. Search Strategy

LB searched Ovid Medline, PubMed, Web of Science, Embase, Scopus, and EBSCOhost (PsycINFO, CINAHL) from 2022 to 2024. The search was conducted between May and June 2024. Search terms are listed in the Supplementary material (Table S1). The search strategy was limited to articles published in the English language only. The reference lists of the included studies were searched and tested against the inclusion criteria to find any additional potential studies. Following the removal of duplicates, titles and abstracts were screened for eligibility by two authors, RN and LB. A third author was available to resolve discrepancies when required (ROS).

### 2.3. Eligibility Criteria

A study was considered for this rapid review if it met the following inclusion criteria (a) investigated physical activity programmes in older adults aged 70 and older; (b) investigated physical activity programmes in older adults where average age > 70 years old and (c) included an intervention programme that included only physical activity as the intervention component. Excluded studies were those which (a) included participants from outside the included age bracket (average age is < 70 years old); (b) were published in languages other than English; (c) were conference abstracts, study protocols, theses or dissertations; (d) were opinion pieces (e) were studies without a relevant population group, i.e., staff reporting how programmes worked; (f) qualitative papers for programme evaluation; (g) were studies developing interventions without participants; (h) secondary analysis papers and (i) studies published before 2022.

### 2.4. Selection of Reviews

Database searches were exported into Excel, and duplicates were removed. Title, abstract and full-text screening were conducted. A pool of three reviewers assessed the eligibility of the primary studies. The two reviewers discussed and attempted to resolve disagreements; a third reviewer intervened when a consensus could not be reached. The PRISMA guidelines will be adhered to improve reporting transparency (Table S2). Data was extracted using a standardised Excel form by two reviewers. A third reviewer verified the completeness of the extracted data, and a third reviewer resolved any discrepancies or disagreements. The information extracted included type of study, number of participants, outcome measures, details on the intervention (setting, duration, frequency, providers/resources, theoretical framework, content, and control), study length, significant findings, and risk of bias.

### 2.5. Methodological Quality Assessment

The Assessment of Multiple Systematic Reviews version 2 (AMSTAR2) checklist, comprising 16 items, was used by two reviewers (RN and ROS) to independently assess the quality of the methods reported in the studies (e.g., comprehensive search strategy, risk of bias, heterogeneity, etc.). This valid and reliable tool includes ratings for quality of the study design, method of analysis, reporting of results, and risk of bias [24]. The AMSTAR 2 score is categorised as high in studies that have no or one non-critical weakness, moderate with more than one non-critical weakness, low when the study has only one serious flaw without or with non-critical weaknesses, and critically low when a study has more than one essential flaw without or with non-critical weaknesses. Discrepancies between the AMSTARS 2 scores for the articles were resolved by discussion with a third reviewer (LB). Evaluating individual components within the reviews was beyond the scope of this umbrella review.

### 2.6. Data Extraction and Synthesis

Two reviewers extracted the data independently. For each eligible review, we extracted the key study characteristics, including authors, year of publication, journal, population, and outcome examined. We recorded a summary of the appraisal methods and interpretations of their findings. The extracted data were synthesised to address the study’s aims. The data were compiled into a single Microsoft Excel spreadsheet (Microsoft Corporation, Redmond, WA, USA). Results were summarised through descriptive statistics. Due to different outcome measures of each included study, no statistical comparison was completed in this umbrella review.

### 2.7. Deviations from Registered Protocol

Some changes were made to the registered protocol of Prospero (CRD42024521063), and the protocol was updated. This was due to the number of initial search results. The initial protocol examined primary RCTS investigating physical activity interventions in older adults between 2014 and 2024. The decision was made to change the rapid review to a rapid umbrella review on systematic reviews from 2022 to 2024.

## 3. Results

### 3.1. Study Selection

**Figure 1:**
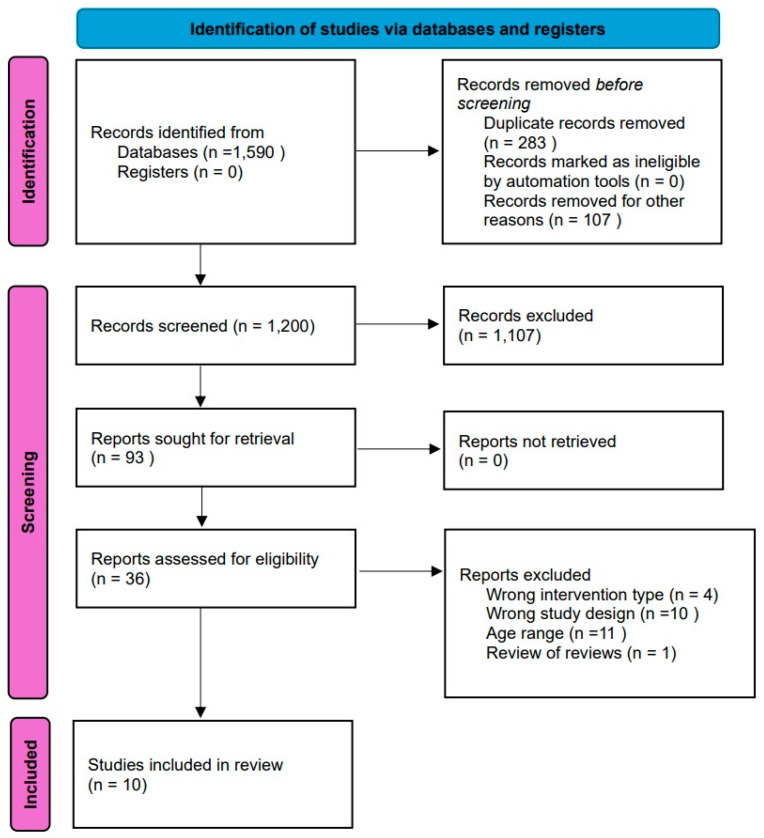
PRISMA flow diagram.

Following the initial database searches, 1590 articles were identified using the search strategy (Figure 1). Duplicates were removed, leaving 93 articles. Titles and abstracts were screened, and 57 articles were removed, leaving 36 articles for full-text review. A total of 26 studies were excluded from the review (Table S3). 10 studies met the inclusion criteria and were included within the rapid umbrella review. Review findings will be discussed based on the PICO search strategy (Population, Intervention, Comparison, and Outcome) [25].

### 3.2. Characteristics of Included Systematic Reviews

The 10 included studies comprised 10 systematic reviews, 8 of which included a meta-analysis (Table 1). Studies were published between 2022 and 2024, encompassing 186 research articles. The study samples in each systematic review ranged from 6 to 1254 participants. The total overall sample size for the 10 included studies was 22,652 participants. All studies had an average age of more than 70 years. Studies examined: fear of falling in community-dwelling older adults [26,27,28], sedentary older adults [29], older adults after hip fractures [30], frail older adults [31], older adults living in LTI and diagnosed with dementia [32], older adults with a clinical diagnosis of Alzheimer’s disease [33], older adults with dementia [34] and older adults with cognitive or without cognitive impairment [35]. While each systematic review examined physical activity interventions, they all focused on how physical activity impacted other health-related outcomes. Six of the studies examined cognition or dementia [27,29,32,33,34,35], three studies examined falls, frailty, or bone conditions (e.g., fractures) [26,30,31], and one examined generic health outcomes [28]. All systematic reviews included only randomised controlled trials.

### 3.3. Intervention Programmes

As shown in Table 1, a range of intervention strategies were used within the included systematic reviews. The most common type of exercise intervention was multicomponent with a range of balance, resistance and aerobic exercises (*n* = 6) [26,28,29,30,32,33], two involved Tai Chi [27,34], one included rhythmic physical activity [35] and another focused on resistance bands training [31]. Interventions were either individualised or in a group setting. Some studies were conducted in community settings, at home, via video consultation, or in person. Caregivers or healthcare professionals supervised some studies; others were unsupervised. Exercise dose varied across the included systematic reviews, with the total duration of the interventions ranging from two weeks to 24 months, and from two to five times a week. Some trials included repetitive activities, with sessions lasting between 15 and 150 min. The duration of each exercise session ranged from 15 to 135 min. Interventions lasted between 4 and 96 weeks. Sessions lasted 30–150 min and were performed at a frequency of two to four times per week, at light to moderate intensity.

### 3.4. Outcome Measures and Study Designs

All studies included in the systematic reviews were randomised controlled trials. Outcome measures varied across studies (Table S3). The most common outcomes explored in the included systematic reviews were cognitive function, physical or motor function, or functional movement. Standard scales included Barthel Index, Mini-Mental State Examination, Montreal Cognitive Assessment, Berg Balance Scale, Geriatric Depression Scale, Walking Speed, Tinetti Assessment Tool Scale, and Timed Up and Go.

All studies used a method of quality appraisal, with some assessing the evidence using two tools. The tools used included the PEDro Scale (*n* = 4) [26,32,33,35], the JBI Critical Appraisal Checklist for RCT studies (*n* = 1) [31], and the Cochrane risk of bias tool for randomised trials (RoB-2) (*n* = 6) [27,28,29,30,31,34]. The quality of evidence was evaluated using the Grading of Recommendations Assessment, Development, and Evaluation (GRADE) (*n* = 3) [28,32,33]. A random effects model was used for all eight studies which conducted a meta-analysis [26,27,28,29,30,31,34,35].

### 3.5. Summary of Intervention Effects

Overall, across the 10 included studies, there were mixed results on whether physical activity interventions would improve outcomes in older adults across various community settings. There was no consistent evidence on which intervention type is the most suitable.

#### 3.5.1. Effects on Falls, Frailty or Bone Conditions

Three studies examined fall, frailty, or bone conditions (e.g., fractures) [26,30,31]. Feng et al. [26] reported no statistically significant effect on subgroups based on type of exercise, duration of interventions, primary or secondary outcome measures, and individual- or group-based exercises. Results from this study showed an overall small to moderate effect size of exercise interventions in reducing the fear of falling.

Zhang et al. [30] reported a significant moderate improvement in overall physical function after a hip fracture compared to the non-exercise group. Meta-regression analysis revealed that the overall result was not substantial in terms of the overall function. Subgroup analysis revealed that neither aerobic nor balance exercise alone had a significant impact on overall physical function. However, the exercise intervention showed a negligible effect on mobility compared to the non-exercise group. Aerobic and resistance exercise alone showed no effect on mobility promotion, but balance exercises improved mobility.

Saragih et al. [31] reported that resistance band exercise reduced frailty after 24 weeks and reduced depression after both 12 weeks and 24 weeks. However, no significant effects were observed for frailty after 12 weeks, and no significant effects were observed for grip strength, leg strength, activities of daily living or quality of life at any time.

#### 3.5.2. Effects on Cognition or Dementia

Six of the studies examined cognition or dementia [27,29,32,33,34,35]. Park et al. [27] reported that Tai Chi and Qigong had small effect sizes on improving cognitive and physical function. Still, no significant differences were found between the effect sizes according to the intervention duration. Zhao et al. [29] reported a significant combined effect, indicating that physical exercise (multi-component and aerobic exercises) can improve cognitive function.

Oliveria et al. [32] reported on the effects of physical exercise on physical, cognitive, and functional capacity, with 10 studies showing mixed results. Multimodal exercise programmes showed mixed results, with 1 study indicating no significant difference in functional performance or cognitive function, while another showed significant effects on functional performance. The subsequent multi-modal intervention examined quality of life and found significant improvements. High Intensity exercise interventions showed mixed results, two studies found no significant difference in depression, functionality, balance and cognitive function, and the final high intensity study indicated a significant difference, with those exercising less likely to suffer moderate/severe lesions in falls. One study looked at strengthening exercises and found significant improvements in physical function. The final intervention type was aerobic exercise programmes. One study found an initial considerable effect on physical and cognitive function, but the authors noted that this effect did not remain after 9 weeks. The final two also reported significant differences in cognitive function.

Oliveria et al. [33] reported a low level of evidence with no effect on mobility, muscle strength, postural balance, cardiorespiratory function, activities of daily living and flexibility with aerobic exercise. The authors reported that multimodal exercises with a shorter overall time offered more benefits to older adults than more extended protocols, e.g., 12 months. Liu et al. [34] reported three studies that showed significant effects on cognitive function for Tai chi interventions but no significant impact on improving physical function or depression symptoms.

Sánchez-Alcalá et al. [35] reported that among the studies assessing depression, six reported statistically significant improvements supporting the rhythmic physical activity-based intervention. In contrast, three studies, utilising the HADS reported that the intervention did not yield statistically significant improvements in this variable. In terms of anxiety, two of the included studies reported statistically significant improvements assessed using the Hospital Anxiety and Depression Scale (HADS). Conversely, two studies did not observe any improvement in anxiety. Overall, a subgroup analysis found a statistically significant median effect favouring interventions based on rhythmic physical activity for GDS-15. The second subgroup analysis, which comprised studies using the Hospital Anxiety and Depression Scale, found a small mean effect that was not statistically significant.

#### 3.5.3. Effects on Generic Health Outcomes

One included review examined generic health outcomes [28]. Nicolson et al. [28] reported no difference between therapeutic exercise and non-exercised comparators on self-reported physical function or performance-based physical function at follow-up. Psychosocial outcomes differed across the included studies. The Geriatric Depression Scale showed no difference in scores at follow-up for a 16-week multicomponent training, whereas a 6-month aerobic exercise programme showed significant improvements on the Philadelphia Geriatric Morale Scale. For falls, two studies reported no difference between groups, while one reported substantial reductions for all falls following a multicomponent home exercise programme. Exercise appeared to reduce the risk of mortality during follow-up.

### 3.6. Summary of Quality and Publication Bias

Overall, studies showed low to moderate quality of evidence with some concerns on bias levels (Table 1). Asymmetric funnel plots indicate possible publication bias [26,27]. Egger’s test was non-significant [29,31]. Egger’s test showed no apparent publication bias [30,34,35].

### 3.7. Critical Appraisal

**Figure 2:**
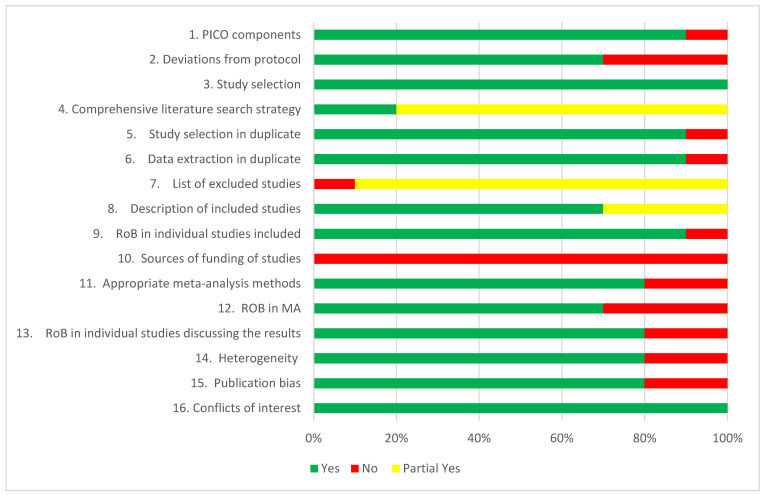
A summary of results in percentage based on AMSTAR 2 Ratings.

AMSTAR-2 ratings revealed four systematic reviews of high quality, one of moderate quality, and five of low quality. Of the reviews, 10% addressed conflicts of interest, 90% included PICO components, and completed study selection. Very few reviews listed the excluded studies or the reasons for exclusion. ROB assessment was addressed by 90% of the reviews. However, two studies did not conduct a meta-analysis. Publication bias was addressed by 80% of reviews. No studies detailed the funding sources of all included studies (Table 2 and Figure 2).

### 3.8. Heterogeneity

Two studies did not assess heterogeneity, and no meta-analysis was conducted. Three studies showed no to low heterogeneity, one showed moderate heterogeneity, and four showed substantial heterogeneity.

## 4. Discussion

### 4.1. Summary of Findings

This is the first umbrella review to investigate the effects of physical activity programmes for older adults aged 70 and above. Globally, there are approximately 90 million people aged 70 and above, which is expected to continue rising due to increased life expectancy and a declining birth rate. Existing evidence highlights the importance of physical activity (PA) as a vital component in maintaining and enhancing health, as well as combating poor health as we age. The findings of this review, along with the increasing number of people aged 70 and above, underscore the importance of and emphasises the need for a more comprehensive understanding of the specific PA requirements for individuals aged 70 and the benefits of physical activity programmes that incorporate aerobic, muscle-strengthening, and balance exercises at least 2 days a week to support active ageing.

Our umbrella review identified 10 systematic reviews, eight of which conducted a meta-analysis. AMSTAR-2 ratings revealed four high-quality systematic reviews, one moderate-quality review, and five low-quality reviews. Based on these AMSTAR 2 ratings, the methodological flaws in the low-quality reviews suggest that higher-quality reviews are needed. These flaws, such as failing to report sources of funding, not using a comprehensive search strategy, or not including a rationale for excluded studies, increase the risk of bias and reduce the reliability of the findings. In particular, the lack of a comprehensive search strategy may increase the risk of publication bias and omission of relevant studies. Similarly, the lack of justification for excluding the studies can undermine the objectivity of the review process. Finally, the lack of discussion around funding sources can skew meta-analysis results and introduce bias in the results or study inclusion. Overall, studies showed low to moderate quality of evidence, with some concerns regarding bias levels, as indicated by Egger’s test.

Nevertheless, findings demonstrated that the role of physical activity interventions for older adults has been explored across an impressive number of outcomes, covering a wide range of issues such as fear of falling, frailty/bone conditions, cognition or dementia, and general health outcomes such as depression. The one study on generic health outcomes [28] reported no difference between therapeutic and non-exercised comparators in physical function at follow-up; however, a significant improvement was observed with aerobic exercise in Geriatric Morale. The three studies [26,30,31] examining falls, frailty, or bone conditions reported on individual and group-based exercises, with no significant effects; however, balance and resistance exercises showed some improvement in frailty and mobility. The six studies [27,29,32,33,34,35] examining cognition or dementia showed that multicomponent or aerobic exercise, as well as rhythmic physical activity interventions, resulted in improvements in cognitive function, suggesting that this type of intervention may be beneficial for individuals over the age of 70.

Across the 10 included studies, there was mixed evidence on whether physical activity interventions could improve outcomes in older adults across various settings, including home, community, and residential settings. There was no consistent evidence on which intervention type is the most suitable (e.g., multicomponent, aerobic, resistance). However, studies on aerobic exercises have suggested significant improvements in physical or cognitive function [29,30,32], but the evidence is inconclusive regarding the duration of this effect. Additionally, the heterogeneity of the included reviews was moderate to high, indicating variability in the intervention effects among the studies. Therefore, significant differences could be overlooked or underestimated. We have presented the results for different groups of the older adult population with cognitive impairments, frailty, or just living in a community dwelling. These findings could be valuable for developing and planning activity programmes and interventions for older adults in future research, including an increased focus on muscle strengthening activities [36]. Overall, due to the variability of the evidence and the level of detail included, the results are inconclusive, and unfortunately, the findings do not provide a definitive conclusion.

### 4.2. Practical Implications and Future Research

This rapid umbrella review provides a first attempt at synthesising reviews on physical activity interventions for adults aged 70 and above. The review could serve as a helpful starting point for future research or an evidence base to inform the development of practice and policy. Our review highlighted several gaps within the current literature on physical activity programmes for adults aged 70 and older. The review highlights the need for high-quality RCTs evaluating the effects of physical activity programmes for older adults aged 70+ and identifies the most suitable intervention for this age group. Future randomised controlled trials and systematic reviews should examine the long-term effects of physical activity on older adults, the impact of physical activity on their loneliness and cognitive function, and focus on which types of intervention programmes work best in this age group, as well as whether group or individual activities yield different results. Very few of the included studies within the systematic reviews discussed the intensity in detail; therefore, intensity should be examined further. While research with older adults can be challenging, tackling these and moving forward with research is essential.

### 4.3. Strengths and Limitations

Conducting an umbrella review on physical activity programmes for older adults (aged > 70 years) has many strengths and limitations. A rapid umbrella review is a timely approach, with the results of this review providing relevant evidence that can be used to strengthen public health policies and systems for older adults. Umbrella reviews are beneficial as they are a valuable research resource involving a systematic and standardised assessment of evidence in a broad area of research [21,37].

A strength of the review conducted was adhering to the review protocol, which also increased the likelihood of capturing all relevant studies through a solid search strategy across electronic databases. The strict criteria in the final selection of the searched literature, along with adherence to the registered protocol, ensured a high-quality methodology. The data screening, extraction, and quality assessment were conducted by two reviewers, with any disagreements resolved by a third reviewer. We critically appraised the evidence in each included systematic review and explored levels of heterogeneity across the included studies.

Several limitations should be acknowledged in this umbrella review, as the original studies have limitations and a paucity of data, with the quality of evidence dependent on the quality of the reviews [21,37]. The inclusion of low-quality studies may also be limiting due to their impact on the credibility of the results. The systematic reviews also varied significantly in their outcome measures, making comparisons challenging. Another limitation of the review is that the included articles were primarily studies published in English, potentially missing relevant articles in other languages. Given the growing evidence base for physical activity interventions and research on older adults in countries such as Spain, China, or Japan, future reviews should be conducted with additional searches for non-English publications. No statistical analysis was performed due to the range of outcomes and types of interventions included, which would make comparison challenging through this method.

## 5. Conclusions

In conclusion, although physical interventions in older adults have been extensively studied for several outcomes and show promise, definitive and conclusive findings cannot be drawn from this review, as the results are not yet definitive and remain inconclusive. The findings of this review, along with the scarcity of studies in the population aged 70 and above, underscore the need for more high-quality randomised controlled trials (RCTs) examining the impact of physical activity on health outcomes as we age. Furthermore, this review emphasises the need for a more comprehensive understanding of the specific PA requirements for individuals aged 70 and above. Based on these AMSTAR 2 ratings, the methodological flaws in the low-quality reviews suggest that higher-quality reviews are needed. In undertaking this review, it became evident that connected factors, such as social isolation, loneliness, and nutrition, are underrepresented in RCTs within the 70+ population, and how physical activity programmes can impact these critical risk factors for active and healthy ageing. Future research should also examine the broader implementation of such programmes.

## Figures and Tables

**Table 1:** Review findings.

Authors	Objectives	No. Included Studies	Year Range of Included Studies	Sample Size	Physical Activity Intervention	Country of Origin of Included Studies	Study Quality
Feng et al. [26]	This review aimed to describe FOF exercise interventions in community-dwelling older adults, evaluate the extent to which these interventions followed the exercise principles and reported exercise parameters, and quantify the effect of these interventions on reducing FOF.	75	1997–2019	12,616	Multi-component exercise interventions, balance exercises, 3-dimensional exercises, strengthening exercises, aerobic exercises, and novel exercises	Japan, Brazil, Sweden, USA, Italy, Greece, Serbia, Spain, Australia, UK, Thailand, Canada, New Zealand, Switzerland, France, Germany, Korea, Austria, Finland, Norway, Taiwan, China, Netherlands	PEDro scale: Range 4–8. 47 rated as good 27 rated as fair 1 rated as poor
Oliveria et al. [33]	To investigate the effects of physical exercise on improving functional capacity in older adults living with Alzheimer’s disease	13	Inception until January 2021	811	Multimodal exercises, including used. Both aerobic and resistance training were employed.	Not included	PEDro scale: Range 4–8.
Saragih et al. [31]	Aimed to examine the effects of resistance band exercises for improving outcomes in frail older adults.	15	2003–2020	1,294	Resistance bands	Japan, Spain, South Korea, China, Italy, the Netherlands, Norway, Portugal, Taiwan and Turkey	PEDro scale: Range 4–8.
Park et al. [27]	The objective of this systematic review was to determine the effects of TCQ on cognitive and physical functions in older adults using meta-analysis, and to determine the impact of TCQ on cognitive function while controlling for physical function using a meta-regression approach.	17	2010–2022	2,235	Tai Chi and Qigong	United States, Hong Kong, China, Taiwan, Thailand, New Zealand,	Cochrane Risk of Bias: 6 rated as low risk 11 rated as some concerns.
Zhang et al. [30]	To investigate the effects of different exercise components on physical function and mobility in adults after hip fracture.	15	2008–2019	1198	Aerobic exercise only, resistance exercise only, and various combinations of aerobic, resistance, functional, and/or balance exercise.	Not listed	Cochrane Risk of Bias: Low risk of bias for most studies
Zhao et al. [29]	To evaluate the effects of physical activity on cognition among sedentary older adults.	7	2009–2020	321	Multi-component exercise intervention and aerobic exercise studies	USA, Brazil, France, Italy and Canada.	Cochrane Risk of Bias: General Quality High
Oliveria et al. [32]	The purpose of this study was to perform a systematic review of the effects of different interventions with physical exercise on the physical, functional And cognitive capacity in institutionalised older adults with dementia.	10	2015–2019	876	Multicomponent exercises, high-intensity functional training and activities of daily living training.	Not listed	GRADE: Low to moderate quality of evidence is present in the included studies. PEDro Scale: Range 5–8
Liu et al. [34]	To assess the effectiveness of Tai Chi in improving cognitive, physical, and emotional function among PWDs.	7	2012–2019	616	Tai Chi	China, UK	Cochrane Risk of Bias: A mix of low to high bias. No study is free from bias.
Nicolson et al. [28]	To evaluate the effects of therapeutic exercise on physical and psychosocial outcomes in community-dwelling adults aged 80 years or older.	16	1997–2020	1660	Multicomponent included resistance training, followed by resistance training, aerobic exercise, functional training and 3d exercise.	Northern Europe or Australia/New Zealand	Cochrane Risk of Bias: 1 rated as low risk of bias 10 rated as some concerns 5 rated as high risk of bias GRADE: Very low to moderate quality of evidence
Sanchez-Alcala et al. [35]	This study aims to identify the effects of rhythmic physical activity interventions on mental health. And quality of life in older adults, with or without mild cognitive impairment.	11	2013–2023	1025	Rhythmic physical activity-based	China and Malaysia, with additional studies from Greece, Spain, Canada, and the United States and Switzerland.	PEDro scale: Range 4–8.

**Table 2:** Results of the AMSTAR checklist items.

AMSTAR Item (Critical Domains in Bold)	Feng et al. [26]	Park et al. [27]	Nicolson et al. [28]	Zhao et al. [29]	Zhang et al. [30]	Saragih et al. [31]	Oliveira et al. [32]	Oliveria et al. [33]	Liu et al. [34]	Sánchez-Alcalá et al. [35]
Did the research questions and inclusion criteria for the review include the components of PICO?	Yes	Yes	Yes	Yes	Yes	Yes	Yes	Yes	No	Yes
2. Did the report of the review contain an explicit statement that the review methods were established prior to the conduct of the review and did the report justify any significant deviations from the protocol?	Yes	Yes	Yes	No	No	Yes	Yes	Yes	No	Yes
3. Did the review authors explain their selection of the study designs for inclusion in the review?	Yes	Yes	Yes	Yes	Yes	Yes	Yes	Yes	Yes	Yes
4. Did the review authors use a comprehensive literature search strategy?	Partial Yes	Partial Yes	Yes	Partial Yes	Partial Yes	Partial Yes	Partial Yes	Yes	Partial Yes	Partial Yes
5. Did the review authors perform study selection in duplicate?	Yes	No—not stated	Yes	Yes	Yes	Yes	Yes	Yes	Yes	Yes
6. Did the review authors perform data extraction in duplicate?	Yes	No—not stated	Yes	Yes	Yes	Yes	Yes	Yes	Yes	Yes
7. Did the review authors provide a list of excluded studies and justify the exclusions?	Partial Yes	No	Partial Yes	Partial Yes	Partial Yes	Partial Yes	Partial Yes	Partial Yes	Partial Yes	Partial Yes
8. Did the review authors describe the included studies in adequate detail?	Yes	Partial Yes	Partial Yes	Yes	Yes	Yes	Yes	Yes	Yes	Partial Yes
9. Did the review authors use a satisfactory technique for assessing the risk of bias (RoB) in individual studies that were included in the review?	Yes	Yes	Yes	Yes	Yes	Yes	No	Yes	Yes	Yes
10. Did the review authors report on the sources of funding for the studies included in the review?	No	No	No	No	No	No	No	No	No	No
11. If meta-analysis (MA) was performed did the review authors use appropriate methods for statistical combination of results?	Yes	Yes	Yes	Yes	Yes	Yes	No MA	No MA	Yes	Yes
12. If meta-analysis was performed, did the review authors assess the potential impact of RoB in individual studies on the results of the meta-analysis or other evidence synthesis?	Yes	Yes	Yes	Yes	Yes	Yes	No MA	No MA	Yes	No
13. Did the review authors account for RoB in individual studies when interpreting/ discussing the results of the review?	Yes	Yes	Yes	Yes	Yes	Yes	No MA	No MA	Yes	Yes
14. Did the review authors provide a satisfactory explanation for, and discussion of, any heterogeneity observed in the results of the review?	Yes	Yes	Yes	Yes	Yes	Yes	No MA	No MA	Yes	Yes
15. If they performed quantitative synthesis did the review authors carry out an adequate investigation of publication bias (small study bias) and discuss its likely impact on the results of the review?	Yes	Yes	Yes	Yes	Yes	Yes	No MA	No MA	Yes	Yes
16. Did the review authors report any potential sources of conflict of interest, including any funding they received for conducting the review?	Yes	Yes	Yes	Yes	Yes	Yes	Yes	Yes	Yes	Yes
**AMSTAR 2 QUALITY SCORE**	High	Low	High	Low	Low	High	Low	Moderate	Low	High

## Data Availability

This study did not create or analyse new data, and data sharing does not apply to this article.

## Supplementary Materials

The following supporting information can be downloaded at https://www.mdpi.com/article/10.3390/geriatrics10040098/s1, Table S1: Suggested search terms. Table S2: PRISMA checklist. Table S3: Excluded Studies.
