# Supplementary material for: Moving as We Age: Effects of Physical Activity Programmes on Older Adults—An Umbrella Review
Source: Geriatrics (Basel). 2025 Jul 23;10(4):98. doi: 10.3390/geriatrics10040098 (PMC12385685; doi:10.3390/geriatrics10040098)
Supplement: Supplementary file 1 [file geriatrics-10-00098-s001.zip › geriatrics-3598275-supplementary.pdf]

Table S1. Suggested search terms

Description	Search terms
Population: Older adults	“old”[tiab] OR “older”[tiab] OR “aged”[tiab] OR “aging”[tiab] OR “ageing”[tiab] OR “elderly” [tiab] Elders” [tiab] OR “Elder”
Outcome: Physical activity	“Activities of daily living”[tiab] OR “Aerobic activities”[tiab] OR “Aerobic activity”[tiab] OR “Aerobic exercise” [tiab] OR “Balance training”[tiab] OR “Physical fitness” OR “Cardiovascular activities”[tiab] OR “Cardiovascular activity”[tiab] OR “OR “Endurance activities”[tiab] OR “Endurance activity”[tiab] OR “Exercise”[mh] OR “Exercise”[tiab] OR “Free living activities”[tiab] OR “Free living activity”[tiab] OR “Functional training”[tiab] OR “Lifestyle activities”[tiab] OR “Lifestyle activity”[tiab] OR “Physical activity”[tiab] OR “Qigong”[tiab] OR “Recreational activities”[tiab] OR “Recreational activity”[tiab] OR “stretching”[tiab] OR “Tai ji”[mh] OR “Yoga”[mh] OR “Qigong”[mh]) OR “Physical activities”[tiab] OR “Physical conditioning”[tiab] OR “Resistance training”[tiab] OR “strength training”[tiab] OR “Tai chi”[tiab] OR “Tai ji”[tiab] OR “Walk”[tiab] OR “Walking”[tiab] OR “Yoga”[tiab] OR “Swimming” [tiab] OR “Hiking” [tiab] OR “Leisure-domain activity” [tiab] OR “muscle stretching activities” OR “running” “physical conditioning” OR “Motor Activity” OR “movement”
Limit: Date	2004-2014
Limit: Language	(English[lang])
Limit: Publication type	NOT (“comment” [Publication Type] OR “thesis” [Publication Type] OR “dissertation” [Publication Type]) OR “editorial” [Publication Type])

Table S2. PRISMA checklist

Section and Topic	Item #	Checklist item	Location where item is reported
**TITLE**			
Title	1	Identify the report as a systematic review.	Page 1
**ABSTRACT**			
Abstract	2	See the PRISMA 2020 for Abstracts checklist.	Page 2
**INTRODUCTION**			
Rationale	3	Describe the rationale for the review in the context of existing knowledge.	Pages 2 and 4
Objectives	4	Provide an explicit statement of the objective(s) or question(s) the review addresses.	Page 4
**METHODS**			
Eligibility criteria	5	Specify the inclusion and exclusion criteria for the review and how studies were grouped for the syntheses.	Pages 5 and 6
Information sources	6	Specify all databases, registers, websites, organisations, reference lists and other sources searched or consulted to identify studies. Specify the date when each source was last searched or consulted.	Page 5
Search strategy	7	Present the full search strategies for all databases, registers and websites, including any filters and limits used.	S1
Selection process	8	Specify the methods used to decide whether a study met the inclusion criteria of the review, including how many reviewers screened each record and each report retrieved, whether they worked independently, and if applicable, details of automation tools used in the process.	Pages 5 and 6
Data collection process	9	Specify the methods used to collect data from reports, including how many reviewers collected data from each report, whether they worked independently, any processes for obtaining or confirming data from study investigators, and if applicable, details of automation tools used in the process.	Pages 6 and 7
Data items	10a	List and define all outcomes for which data were sought. Specify whether all results that were compatible with each outcome domain in each study were sought (e.g. for all measures, time points, analyses), and if not, the methods used to decide which results to collect.	Pages 5-7
	10b	List and define all other variables for which data were sought (e.g. participant and intervention characteristics, funding sources). Describe any assumptions made about any missing or unclear information.	Pages 5-7
Study risk of bias assessment	11	Specify the methods used to assess risk of bias in the included studies, including details of the tool(s) used, how many reviewers assessed each study and whether they worked independently, and if applicable, details of automation tools used in the process.	Page 6
Effect measures	12	Specify for each outcome the effect measure(s) (e.g. risk ratio, mean difference) used in the synthesis or presentation of results.	Page 7
Synthesis methods	13a	Describe the processes used to decide which studies were eligible for each synthesis (e.g. tabulating the study intervention characteristics and comparing against the planned groups for each synthesis (item #5)).	Page 7
	13b	Describe any methods required to prepare the data for presentation or synthesis, such as handling of missing summary statistics, or data conversions.	Page 7

Section and Topic	Item #	Checklist item	Location where item is reported
	13c	Describe any methods used to tabulate or visually display results of individual studies and syntheses.	Page 7
	13d	Describe any methods used to synthesize results and provide a rationale for the choice(s). If meta-analysis was performed, describe the model(s), method(s) to identify the presence and extent of statistical heterogeneity, and software package(s) used.	Page 7
	13e	Describe any methods used to explore possible causes of heterogeneity among study results (e.g. subgroup analysis, meta-regression).	Page 6
	13f	Describe any sensitivity analyses conducted to assess robustness of the synthesized results.	Page 6
Reporting bias assessment	14	Describe any methods used to assess risk of bias due to missing results in a synthesis (arising from reporting biases).	Page 6
Certainty assessment	15	Describe any methods used to assess certainty (or confidence) in the body of evidence for an outcome.	Page 6
**RESULTS**			
Study selection	16a	Describe the results of the search and selection process, from the number of records identified in the search to the number of studies included in the review, ideally using a flow diagram.	Page 7
	16b	Cite studies that might appear to meet the inclusion criteria, but which were excluded, and explain why they were excluded.	Page 7, Fig 1 and S1
Study characteristics	17	Cite each included study and present its characteristics.	Pages 8-9 and Table 1
Risk of bias in studies	18	Present assessments of risk of bias for each included study.	Pages 16 and 17 Figure 2 and Table 2
Results of individual studies	19	For all outcomes, present, for each study: (a) summary statistics for each group (where appropriate) and (b) an effect estimate and its precision (e.g. confidence/credible interval), ideally using structured tables or plots.	Pages 13-16
Results of syntheses	20a	For each synthesis, briefly summarise the characteristics and risk of bias among contributing studies.	Pages 13-17
	20b	Present results of all statistical syntheses conducted. If meta-analysis was done, present for each the summary estimate and its precision (e.g. confidence/credible interval) and measures of statistical heterogeneity. If comparing groups, describe the direction of the effect.	N/A
	20c	Present results of all investigations of possible causes of heterogeneity among study results.	Page 18
	20d	Present results of all sensitivity analyses conducted to assess the robustness of the synthesized results.	N/A
Reporting biases	21	Present assessments of risk of bias due to missing results (arising from reporting biases) for each synthesis assessed.	Page 17
Certainty of evidence	22	Present assessments of certainty (or confidence) in the body of evidence for each outcome assessed.	Pages 13-18
**DISCUSSION**			

Section and Topic	Item #	Checklist item	Location where item is reported
Discussion	23a	Provide a general interpretation of the results in the context of other evidence.	Pages 18-19
	23b	Discuss any limitations of the evidence included in the review.	Page 20
	23c	Discuss any limitations of the review processes used.	Page 20
	23d	Discuss implications of the results for practice, policy, and future research.	Page 19
**OTHER INFORMATION**			
Registration and protocol	24a	Provide registration information for the review, including register name and registration number, or state that the review was not registered.	Pages 4, 7 and 21
	24b	Indicate where the review protocol can be accessed, or state that a protocol was not prepared.	Page
	24c	Describe and explain any amendments to information provided at registration or in the protocol.	Page 7
Support	25	Describe sources of financial or non-financial support for the review, and the role of the funders or sponsors in the review.	Page 22
Competing interests	26	Declare any competing interests of review authors.	Page 22
Availability of data, code and other materials	27	Report which of the following are publicly available and where they can be found: template data collection forms; data extracted from included studies; data used for all analyses; analytic code; any other materials used in the review.	S1 and S2

Page, M.J.; McKenzie, J.E.; Bossuyt, P.M.; Boutron, I.; Hoffmann, T.C.; Mulrow, C.D, et al. The PRISMA 2020 statement: an updated guideline for reporting systematic reviews. BMJ **2021**;372:n71. https://doi.org/10.1136/bmj.n71

Table S3. Excluded Studies

sTable	Excluded studies, with reasons for exclusion	Reason for exclusion
1	A systematic review and meta-analysis: Assessment of hospital walking programs among older patients	Study design
2	Association between physical activity measured by accelerometry and cognitive function in older adults: A systematic review	Study design
3	Associations of objectively measured physical activity and sedentary behaviour with fall-related outcomes in older adults: A systematic review	Study design
4	Does High-Velocity Resistance Exercise Elicit Greater Physical Function Benefits Than Traditional Resistance Exercise in Older Adults? A Systematic Review and Network Meta-Analysis of 79 Trials.	Intervention type
5	Effect of eHealth-delivered exercise programmes on balance in people aged 65 years and over living in the community: a systematic review and meta-analysis of randomised controlled trials	Intervention type
6	Effect of Exercise Cognitive Combined Training on Physical Function in Cognitively Healthy Older Adults: A Systematic Review and Meta-Analysis	Intervention type
7	Effect of exercise on the cognitive function of older patients with type 2 diabetes mellitus: A systematic review and meta-analysis	Intervention type
8	Effect of Physical Exercise on Cognitive Function of Alzheimer's Disease Patients: A Systematic Review and Meta-Analysis of Randomized Controlled Trial	Age range
9	Effectiveness of exercise interventions on fall prevention in ambulatory community-dwelling older adults: a systematic review with narrative synthesis	Age range
10	Effectiveness of exercise rehabilitation interventions on depressive symptoms in older adults post hip fracture: a systematic review and meta-analysis	Study design
11	Effectiveness of home-based exercise delivered by digital health in older adults: a systematic review and meta-analysis	Age range
12	Effectiveness of interventions to prevent pre-frailty and frailty progression in older adults evidence	Study design
13	Effects of Home-Based Exercise Programs on Mobility, Muscle Strength, Balance, and Gait in Community-Dwelling Older Adults: A Systematic Review and Meta-Analysis	Age range
14	Exercise for acutely hospitalised older medical patients	Study design
15	Impact of exercise training on muscle mitochondria modifications in older adults: a systematic review of randomized controlled trials	Age range
16	Influence of different modes of exercise training on inflammatory markers in older adults with and without chronic diseases: A systematic review and meta-analysis	Age range
17	Personally tailored exercises for improving physical outcomes for older adults in the community: A systematic review	Study design
18	Physical-activity interventions to reduce fear of falling in frail and pre-frail older adults: a systematic review of randomized controlled trials	Age range

19	Role of Resistance Training in Mitigating Risk for Mobility Disability in Community-Dwelling Older Adults: A Systematic Review and Meta-analysis	Age range
20	The effect of dance on physical health and cognition in community dwelling older adults: A systematic review and meta-analysis	Age range
21	The effect of different exercise programs on sarcopenia criteria in older people: A systematic review of systematic reviews with meta-analysis	Study design
22	The effectiveness of a group-based Otago exercise program on physical function, frailty and health status in older nursing home residents: A systematic review and meta-analysis	Study design
23	The effectiveness of Otago exercise program in older adults with frailty or pre-frailty: A systematic review and meta-analysis	Study design
24	The effects of different types of Tai Chi exercises on preventing falls in older adults: a systematic review and network meta-analysis	Age range
25	The Effects of High-Speed Resistance Training on Health Outcomes in Independent Older Adults: A Systematic Review and Meta-Analysis	Study design
26	The impact of Otago exercise programme on the prevention of falls in older adult: A systematic review	Age range
